# Long‐term effect of caesarean section on the gut microbial taxonomical profile and metabolic function of children at pre‐school age

**DOI:** 10.1002/ctm2.1470

**Published:** 2023-11-06

**Authors:** Chang Gao, Dan‐Tong Shao, Cheng‐Rui Wang, Ya‐Shu Kuang, Jin‐Hua Lu, Ding‐Yuan Zeng, Jian‐Rong He, Xiu Qiu

**Affiliations:** ^1^ Division of Birth Cohort Study Guangzhou Women and Children's Medical Center, Guangzhou Medical University Guangzhou Guangdong China; ^2^ Department of Women's Health Guangdong Provincial Key Clinical Specialty of Women and Child Health, Guangzhou Women and Children's Medical Center Guangzhou Medical University Guangzhou Guangdong China; ^3^ Liuzhou Maternity and Child Healthcare Hospital Affiliated Women and Children's Hospital of Guangxi University of Science and Technology Liuzhou China; ^4^ Guangdong Provincial Clinical Research Center for Child Health Guangzhou Women and Children's Medical Center Guangzhou Medical University Guangzhou Guangdong China; ^5^ Provincial Key Laboratory of Research in Structure Birth Defect Disease and Department of Pediatric Surgery Guangzhou Women and Children's Medical Center Guangzhou Medical University Guangzhou Guangdong China

To the Editor:

In this study, we observed the long‐term effect of caesarean section (CS) on the gut microbiome of pre‐school age children, both on the microbial taxonomical profile and metabolic function. Interestingly, taxonomical differences due to CS were mostly found in children of younger age, but the microbial functional alterations were observed in children of older age.

Gut microbiome is constantly evolving and adapting to the surrounding environments, especially during the early months of life when the microbial composition is undergoing turbulent changes.^1^ The gut microbiome of children tends to stabilise after 3 years of age,^2^ but continues to mature slowly thereafter.^3^ To date, only limited studies^3^, ^4^ have investigated the effect of CS on gut microbial profile of children beyond infancy (up to 12 months). These studies employed fluorescence in situ^4^ or 16S rRNA^3^ for microbial analysis, which were insufficient to explore microbial functions.

We conducted this study with metagenomic sequencing method to examine the effect of CS on the gut microbiome of children of pre‐school age. Between March and April 2017, 2199 children were recruited from 16 public kindergartens in Guangzhou, China. Faecal samples were collected and randomly selected (*n* = 1104) for gut microbial analysis. Among these children, 1034 with valid data regarding their parental, perinatal and birth characteristics derived from parent‐administered questionnaires were included in this analysis. All participants provided written consents, and this study was approved by Guangzhou Women and Children's Medical Center ethic committee. Detailed methodologies regarding the study design, microbial analysis for faecal samples and statistical analysis are available in Supporting Information.

Forty‐three percent of the participants were delivered via CS (Table 1). CS‐born children had higher weight *Z*‐score at birth (.30 vs. .07, *p* < .001) than those delivered vaginally. Mothers who gave birth via CS were significantly elder at sampling (33 vs. 32 years, *p* < .001), had higher pre‐pregnancy body mass index (BMI) (*p* < .001) than those who underwent vaginal delivery.

**TABLE 1:** Demographic characteristics of study population.

	Vaginal delivery		Caesarean section		
	(*n* = 584)		(*n* = 450)		*p*‐Value^a^
Characteristics of children					
Sex, No. (%)					.330
Male	342	(58.56)	260	(57.78)	
Female	242	(41.44)	190	(42.22)	
Age, mean (SD), days	1935	(295)	1880	(318)	.006
Gestational age at birth, mean (SD), weeks	38.80	(1.60)	38.60	(1.60)	.011
Birth weight *Z*‐score, mean (SD)	.07	(.99)	.30	(1.05)	<.001
Ever breastfeeding, No. (%)					.360
Yes	538	(92.12)	403	(89.56)	
No	46	(7.88)	46	(10.22)	
Feeding practices within first 6 months, No. (%)					.030
Exclusive BM	224	(38.36)	162	(36.00)	
Dominant BM	195	(33.39)	119	(26.44)	
Dominant IF	77	(13.18)	81	(18.00)	
Exclusive IF	84	(14.38)	86	(19.11)	
Maternal characteristics					
Maternal age at sampling, mean (SD), years	32.00	(5.43)	33.30	(4.57)	<.001
Maternal education, No. (%)					<.001
Secondary school or below	215	(36.82)	112	(24.89)	
Senior or technical secondary school	151	(25.86)	136	(30.22)	
College or beyond	204	(34.93)	194	(43.11)	
Maternal pre‐pregnancy BMI, mean (SD), kg/m^2^	20.70	(2.53)	21.50	(2.99)	<.001
Maternal BMI group, No. (%)					<.001
Underweight	117	(20.03)	67	(14.89)	
Normal weight	421	(72.09)	312	(69.33)	
Overweight or obese	26	(4.45)	59	(13.11)	
Maternal GWG, mean (SD), kg	11.70	(5.16)	12.90	(5.56)	.001
Adequacy of maternal GWG^b^, No. (%)					.003
Inadequate	257	(44.01)	112	(36.67)	
Adequate	153	(26.20)	115	(26.44)	
Excessive	142	(24.32)	209	(33.56)	

Abbreviations: BM, breast milk feeding; BMI, body mass index; GWG, gestational weight gain; IF, infant formula feeding.

^a^ Statistically significant differences were determined by Welch's test (for continuous variables) and chi‐square test (for categorical variables).

^b^ Adequacy of maternal gestational weight gain was determined based on the Institute of Medicine standard, the adequate GWG for women with different weight categories are specified as following: 12.5–18.0 kg for underweight women (BMI < 18.5); 11.5–16.0 kg for normal weight women (BMI ≥ 18.5, <25); 7.0–11.5 kg for overweight women (BMI ≥ 25, <30); 5.0–9.0 for obese women (BMI≥30).

Overall, for the entire population, the top five most abundant microbial phylum were *Actinobacteria, Bacteroidetes, Firmicutes, Proteobacteria* and *Verrucomicrobia* (Figure 1A), together representing nearly 96% of the total microbial composition. CS‐born children scored significantly lower for richness (*p* = .019, FDR *p* = .076, FDR *p =* .110 for adjustment by age and sex; Figure 1B), but were similar to that of those born vaginally regarding other indices. The microbial beta‐diversity of children born via different modes was significantly different after adjustment for age and sex (Figure 1C, Bray–Curtis *p* = .001, Jaccard *p* = .002). Significant enrichment of *Clostridium* spp. was observed in children born via CS as compared to those born vaginally (Figure 1E), and many of these are classified as opportunistic pathogens. *Clostridium bolteae* were found to be enriched in children of autism spectrum disorder (ASD) than in children without it.^5^ *Clostridium ramosum* enhances lipids uptake and hence promotes obesity as demonstrated in the in vitro study.^6^ In addition, several gene segments of *C. bolteae*, *Clostridium clostridioforme* and *C. ramosum* have been related to the antimicrobial resistance in various cultures.^7^ Altogether, these strains might possess adverse health implications on the host.

**FIGURE 1:**
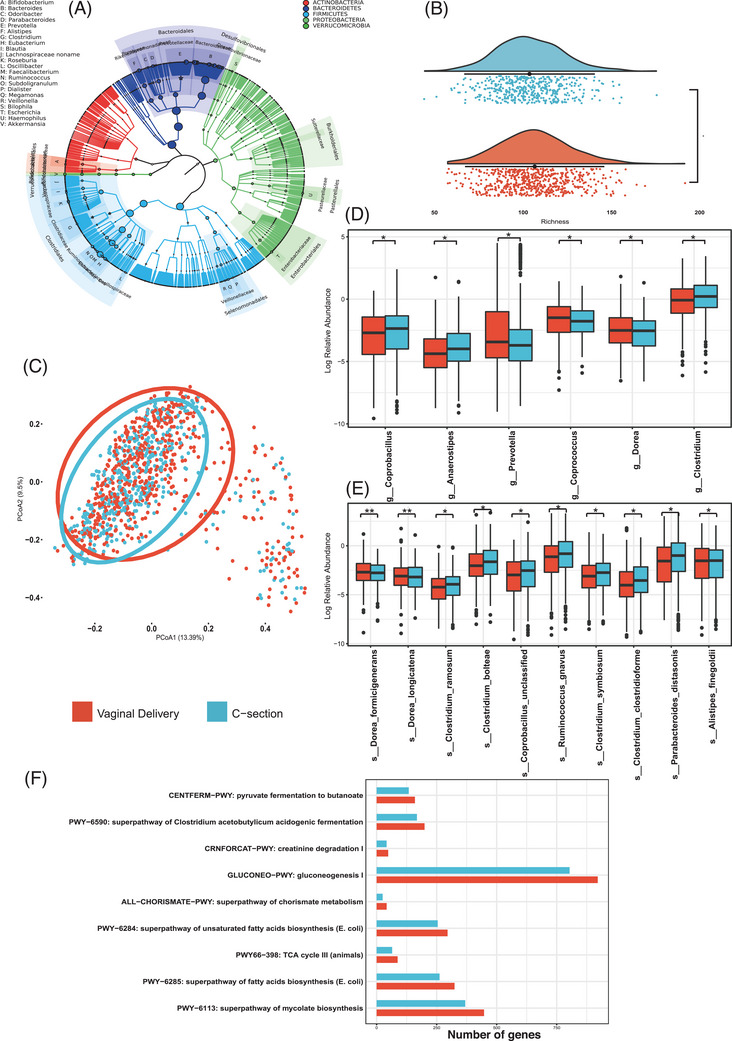
Comparison of gut microbiome between children delivered vaginally and via caesarean section (CS): (A) overall microbial composition of all study population; (B) differences in the number of observed microbial species (richness) between children born via the two different modes as determined by Kruskal Wallis method, *p* = .019; (C) the dissimilarities in gut microbial composition of children born via two different modes as determined by Bray–Curtis method and permutational multivariate analysis of variance, *p* = .001; (D and E) differentially abundant taxa found between children born via two different methods as determined by Wilcoxon test and adjusted by Benjamini and Hochberg method (*p* < .05 for all), at genus and species level, respectively; (F) differential microbial functioning pathways found between children born via the two different modes as determined by analysis of composition of microbes method (ANCOM), displayed as mean of absolute gene counts of the pathways. Colours indicate different modes of delivery, where red represents vaginal delivery and blue represents caesarean section. *Represents .01 < *p* < .05; **represents .001 < *p* < .01.

Stratification by age groups revealed that the microbial beta‐diversity was significantly different between children born via different modes of delivery in those of younger age only (*p* = .037 for age under 5, *p* = .012 for age 5, *p* = .744 for age above 5), although no differences for alpha‐diversity in all age groups (Figure S1). Differences in microbial profile were mainly found at or below 5 years (age ranges were ≥60 and <72 months, <60 months, respectively; Figure S2). Notably, 51 downregulated pathways, mostly related to vitamin metabolism (35%), were found in CS born children aged above 5 (≥72 months), when compared to their vaginally born counterparts (Figure 2E,F; Table S1). It is possible that the children are exposed to more complexed environmental factors and food system over time, which then replaced mode of delivery and became the key determinants of microbial composition.^2^, ^3^ Previous meta‐transcriptomic analysis has shown that the ability of gut microbiome to produce K and B group vitamins are at comparable levels across healthy population,^8^ and these microbiome‐derived vitamins collectively contribute to 30% of our dietary requirement, lack of which might have adverse health consequences on the host.

**FIGURE 2:**
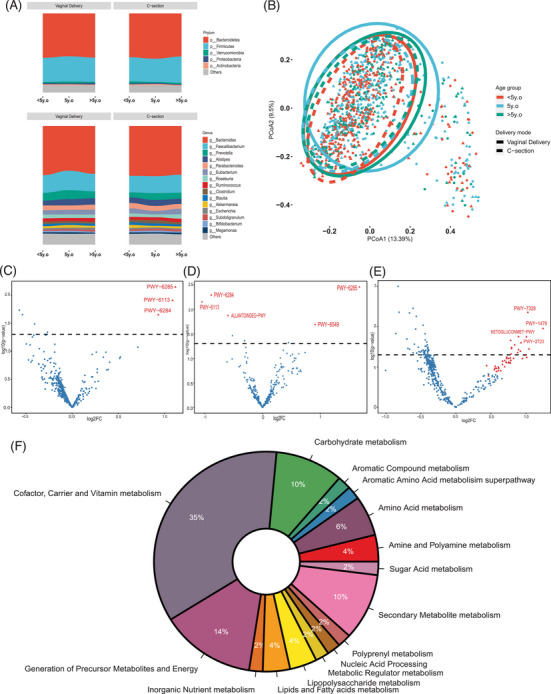
Comparison of microbial profile and metabolic functions of children born via caesarean section (CS) and delivered vaginally in different age groups. (A) The distribution of microbial taxa of children of different age groups and born via two different modes, displayed are the phylum and genus level of taxa; (B) the dissimilarities in gut microbial composition of children born via two different modes as determined by Bray–Curtis method and permutational multivariate analysis of variance, colour indicates different age groups where red represents those under 5 (<60 months), green represents those of 5 years (≥60, <72 months) and blue represents those above age 5 (≥72 months), and the line indicates mode of delivery, where solid line refers to vaginal delivery and dotted line represents CS (*p* = .037 for age under 5; *p* = .012 for age at 5; *p* = .744 for age above 5); (C–E) volcano plot displays the differential microbial functioning pathways found between children born via the two different modes as determined by analysis of composition of microbes method, for those under 5, at 5 and above 5 years of age (only showing top four of 51 pathways), respectively; red solid triangle represents significant difference, where on left side indicates higher abundancy in CS born children and right side refers to higher abundancy in vaginal delivered children; (F) all 51 differentially abundant metabolic pathways of children above 5 years of age (presented in e) categorised by their metabolic functions as indexed by MetaCyc, names and identification numbers of individual pathways are available in Table S1.

It is known that breastfeeding experience shapes the trajectory of gut microbial development,^9^ whether breast milk (BM) could ameliorate the adverse effect of CS on childhood gut microbiota is therefore of interest to explore. Over 60% CS‐delivered children were fed on a predominantly BM diet within their first 6 months of life (Table S2). Infant formula (IF)‐fed CS‐born children scored lowest for gut microbial richness, as compared to either vaginally born or BM‐fed CS‐born children, although no other alpha‐diversity indices were different (Table S3). On the whole population level, it seems that BM feeding ameliorated CS‐induced changes in gut microbial metabolic functions. However, when breakdown by age groups (Tables S4–S6), for children born via same delivery mode, only minor differences in the gut microbial profile and metabolic activities were found to be due to early feeding practices. Our previous finding was largely driven by the younger age group, in whom the effect of BM is likely to be more substantial.^9^

This is the first study to examine the effect of CS on the gut microbiome of children at pre‐school age using metagenomic sequencing. Recall bias was unavoidable due to information collected through questionnaire in a retrospective manner. In addition, information regarding medical history, the use of antibiotics and diet were not collected in this trial, both of which could substantially affect the gut microbiome of children. It must be acknowledged that our samples were derived from a single time point, limiting interpretation of changes or trajectory of gut microbiome of children over time. In addition, our interpretation regarding altered metabolic functions was based on metagenomic sequencing results only. Further study should combine the use of meta‐transcriptomic data to confirm these observations.

In conclusion, we found that CS‐induced microbial change in pre‐school age children is featured by enrichment of *Clostridium* spp. The altered microbial metabolic functions in older children are mostly related to vitamin metabolism, consequences of which remain to be explored.

## CONFLICT OF INTEREST STATEMENT

The authors declare they have no conflicts of interest.

## FUNDING INFORMATION

Key Program of GuangDong Basic and Applied Basic Research Foundation, Grant Number: 2022B1515120080, 2020B1111170001; China Postdoctoral Science Foundation, Grant Number: 2022M710883; National Natural Science Foundation of China, Grant Number: 82173525, 82003471; GuangDong Basic and Applied Basic Research Foundation, Grant Number: 2021A1515110194; Guangzhou Science and Technology Project, Grant Number: 202201020656.

## Supporting information

Supporting Information

Supporting Information

Supporting Information

## Data Availability

The data that support the findings of this study are available on request from the corresponding author. The data are not publicly available due to privacy or ethical restrictions.
