# Supplementary material for: Long‐term effect of caesarean section on the gut microbial taxonomical profile and metabolic function of children at pre‐school age
Source: Clin Transl Med. 2023 Nov 6;13(11):e1470. doi: 10.1002/ctm2.1470 (PMC10626499; doi:10.1002/ctm2.1470)
Supplement: Supplementary file 3 — Supporting Information [file CTM2-13-e1470-s002.docx]

**Title** Long-term effect of cesarean section on the gut microbial taxonomical profile and metabolic function of children at pre-school age

**Author list:** Chang Gao, Ph.D ^a*^, Dan-Tong Shao, Ph.D ^a*^, Cheng-Rui Wang, M.S ^a^, Ya-Shu Kuang, M.S ^a^, Jin-Hua Lu, M.D. ^a,b^, Ding-Yuan Zeng Ph.D ^d^, Jian-Rong He, Ph.D ^a,b#^, Xiu Qiu, Ph.D ^a,b,c,e#^

**Supplemental Materials**

Table of content

**Supplementary Text 1** – Methodological descriptions

**Supplementary Table 1**. Differentially regulated gut microbial metabolic pathways between children born via vaginal delivery and cesarean section.

**Supplementary Table 2**. Demographic characteristics of study population based on mode of delivery and early feeding practices.

**Supplementary Table 3.** The impact of early feeding practices on cesareans section induced microbial compositional and functional alterations in all study participants.

**Supplementary Table 4.** The impact of early feeding practices on cesareans section induced microbial compositional and functional alterations for children under 5 years of age (<60 months).

**Supplementary Table 5.** The impact of early feeding practices on cesareans section induced microbial compositional and functional alterations for children aged five ($\geq$60, <72 months).

**Supplementary Table 6.** The impact of early feeding practices on cesareans section induced microbial compositional and functional alterations for children age above five years old ($\geq$72 months).

**Figure S1.** Comparison of the gut microbial alpha indices (Shannon Wiener Index, Simpson index, Richness and J Pielou Index) between children born vaginally or via cesarean section in different age groups.

**Figure S2.** Comparison of the distribution of previously identified differential microbial taxa between children born virginally and via cesarean section in stratified analysis defined by age.

**Supplementary Text 1 – Methodological descriptions**

**Study design and population**

This cross-sectional study was conducted between June and November 2016, to investigate the growth and development of children of pre-school age in Guangzhou, China to provide timely data for health surveillance purpose. We selected 16 public kindergartens for participant recruitment within the region of Baiyun and Huadu, the topmost and fifth populated districts of Guangzhou, respectively. Between March and April 2017, 2199 children donated fecal samples and we randomly selected that of half participating children (n=1104) for gut microbial profiling. Among these children, 1034 with valid data derived from parent-administered questionnaires were included in the current analysis.

**Exposure**

Parent-administered questionnaire was included in the original project, where information regarding their parental, perinatal and birth characteristics were sought from. Parents were specifically asked for the mode of delivery for being ‘natural vaginal delivery’, ‘assisted vaginal delivery with suction or forceps’ or ‘cesarean section’, we grouped the former two options as vaginal delivery in this analysis.

**Covariates**

Information regarding birth characteristics (length, weight, and gestational age), maternal height, weight within first eight weeks and/or within last month of pregnancy, gestational weight gain (GWG), breastfeeding experience and feeding practices in first six months (exclusive/dominant BM feeding, dominant/exclusive infant formula [IF] feeding) were extracted from the parent administered questionnaire. INTERGROWTH-21^st^ standard (1) was used for newborn birth weight Z-score calculation. Either maternal weight within the first eight weeks of pregnancy or maternal weight within the last month of pregnancy subtracted by GWG was used to calculated maternal pre-pregnancy BMI.

**Stool sample collection and microbiome analysis**

Stool samples were collected and temporarily freezing-stored at home and brought to the attending kindergartens, where shipment is organized to transport all samples directly to BGI (Shenzhen, China) on dry ice. All samples were kept frozen at -80$℃$ until subsequent DNA extraction. Microbial analysis was performed by BGI Shenzhen by using whole-metagenome shotgun sequencing, the detail has been described elsewhere (2, 3). In brief, DNA was extracted using Qiagen QIAamp DNA Stool Mini Kit (Qiagen, Hilden, Germany) and stored at -20$℃$ prior to analysis. Five hundred ng of input DNA was used for library formation and fragmented ultrasonically with Covaris E220 (Covaris, Brighton, UK), yielding 300 to 700 bp of fragments. Sheared DNA without size selection was purified with an Axygen™ AxyPrep™ Mag PCR Clean-Up Kit. An equal volume of beads was added to each sample. We performed end-repairing and A-tailing with a 2:2:1 mixture of T4 DNA polymerase (ENZYMATICS™ P708–1500), T4 polynucleotide kinase (ENZYMATICS™ Y904–1500), and rTaq DNA polymerase (TAKARA™ R500Z). Adaptors with specific barcodes (Ad153 2B) were ligated to the DNA fragment, and PCR amplification was carried out. PCR products was denatured at 95°C and ligated by T4 DNA ligase (ENZYMATICS™ L603-HC-1500) at 37°C to generate a single-strand circular DNA library. Eight barcoded libraries were pooled in equal amounts to make DNA Nanoballs (DNB). Using BGI seq 500 platform, an in-depth sequencing was then performed with 100 base pair lengths. Low quality reads were either removed or trimmed, and potential contamination of human genome was removed by comparing against the KneadData database (kneaddata_db_human_genome), which resulted in an average of 58M reads per sample. The function of microbial genes was annotated according to MetaCyc database. A total number of species and pathways studied were 759 and 539 respectively.

**Statistical Analysis**

Differences in the participants’ characteristics between groups were assessed using Welch’s test for continuous variables and Chi-Square test for categorical variables, respectively. The microbial alpha-diversity was described using observed species, Shannon, Simpson and J Pielou index. The dissimilarities in microbial composition between groups were performed by Bray-Curtis matrix and jaccard matrix and was tested by PERMANOVA adjusted by age and sex using Vegan package in R. Microbial taxa were restricted to with a relative abundance ≥ 0.01% and presented in ≥ 10% of the population and were compared between groups with Wilcoxon test adjusted by Benjamini & Hochberg method. Between-group comparisons for microbial metabolic functions were carried out using ANCOM method (4). Stratified analysis was performed according to three age groups: age under five (< 60 months), age five ($\geq$60, $<$72 months), and age above five ($\geq$72 months). Subgroup analysis was performed to investigate the impact of early feeding practices (categorized as predominantly BM or IF feeding for simplicity) on CS induced microbial alteration in children. All analyses were performed using R (version 4.1.1; Vienna, Austria) and a two-sided *P* < 0.05 was considered statistically significant.

**Table S1.** Differentially regulated gut microbial metabolic pathways between children born via vaginal delivery and cesarean section.

| **Pathway** | **Categorization** | **log2FC** | **log2FC.pval** | **Reject**  **number** |
| --- | --- | --- | --- | --- |
| **Under 5 years old** |  |  |  |  |
| PWY-6285: superpathway of fatty acids biosynthesis (E. coli) | Lipids and Fatty acids metabolism | -1.11996 | 0.0071773 | 492 |
| PWY-6113: superpathway of mycolate biosynthesis | Lipids and Fatty acids metabolism | -1.0884587 | 0.0124496 | 485 |
| PWY-6284: superpathway of unsaturated fatty acids biosynthesis (E. coli) | Lipids and Fatty acids metabolism | -0.9340525 | 0.0223903 | 452 |
| **At 5 years old** |  |  |  |  |
| PWY-7456: mannan degradation | Carbohydrate metabolism | -1.6787415 | 0.0034898 | 505 |
| PWY-3781: aerobic respiration I (cytochrome c) | Generation of Precursor Metabolites and Energy | 1.0498624 | 0.0070366 | 496 |
| PWY-7279: aerobic respiration II (cytochrome c) (yeast) | Generation of Precursor Metabolites and Energy | 0.8885734 | 0.0051075 | 488 |
| PWY-6549: L-glutamine biosynthesis III | Transport | -0.9119417 | 0.0203995 | 435 |
| ALLANTOINDEG-PWY: superpathway of allantoin degradation in yeast | Degradation/Utilization/Assimilation | 0.6000317 | 0.0133733 | 410 |
| **Above 5 years old** |  |  |  |  |
| PWY-2723: trehalose degradation V | Carbohydrate metabolism | 1.008791 | 0.017912 | 428 |
| PWY-7269: NAD/NADP-NADH/NADPH mitochondrial interconversion (yeast) | Cofactor, Carrier and Vitamin metabolism | 0.929825 | 0.025096 | 421 |
| PWY-7328: superpathway of UDP-glucose-derived O-antigen building blocks biosynthesis | Carbohydrate metabolism | 1.027124 | 0.004413 | 419 |
| PWY0-1479: tRNA processing | Nucleic Acid Processing | 1.245379 | 0.011164 | 409 |
| KETOGLUCONMET-PWY: ketogluconate metabolism | Sugar Acid metabolism | 0.895538 | 0.017774 | 407 |
| AST-PWY: L-arginine degradation II (AST pathway) | Amino Acid metabolism | 0.795892 | 0.032966 | 401 |
| PWY-5705: allantoin degradation to glyoxylate III | Amine and Polyamine metabolism | 0.839707 | 0.02845 | 401 |
| ENTBACSYN-PWY: enterobactin biosynthesis | Secondary Metabolite metabolism | 1.044576 | 0.035797 | 400 |
| PWY-6891: thiazole biosynthesis II (Bacillus) | Cofactor, Carrier and Vitamin metabolism | 0.881325 | 0.044096 | 396 |
| PWY-5138: unsaturated, even numbered fatty acid &beta;-oxidation | Lipids and Fatty acids metabolism | 0.792488 | 0.03501 | 395 |
| PWY-6895: superpathway of thiamin diphosphate biosynthesis II | Cofactor, Carrier and Vitamin metabolism | 0.985388 | 0.051145 | 393 |
| UBISYN-PWY: superpathway of ubiquinol-8 biosynthesis (prokaryotic) | Cofactor, Carrier and Vitamin metabolism | 0.788455 | 0.047457 | 393 |
| PWY-5913: TCA cycle VI (obligate autotrophs) | Generation of Precursor Metabolites and Energy | 0.781711 | 0.021897 | 391 |
| PWY0-321: phenylacetate degradation I (aerobic) | Aromatic Compound metabolism | 0.895985 | 0.037247 | 391 |
| PWY-5845: superpathway of menaquinol-9 biosynthesis | Cofactor, Carrier and Vitamin metabolism | 1.012129 | 0.058195 | 388 |
| PWY-5850: superpathway of menaquinol-6 biosynthesis I | Cofactor, Carrier and Vitamin metabolism | 1.012129 | 0.058195 | 388 |
| PWY-5896: superpathway of menaquinol-10 biosynthesis | Cofactor, Carrier and Vitamin metabolism | 1.012129 | 0.058195 | 388 |
| ECASYN-PWY: enterobacterial common antigen biosynthesis | Carbohydrate metabolism | 0.822581 | 0.061314 | 387 |
| PWY-4041: &gamma;-glutamyl cycle | Cofactor, Carrier and Vitamin metabolism | 0.751953 | 0.061757 | 386 |
| PWY-5860: superpathway of demethylmenaquinol-6 biosynthesis I | Cofactor, Carrier and Vitamin metabolism | 0.95043 | 0.061734 | 386 |
| PWY-5862: superpathway of demethylmenaquinol-9 biosynthesis | Cofactor, Carrier and Vitamin metabolism | 0.95043 | 0.061734 | 386 |
| PWY0-1533: methylphosphonate degradation I | Inorganic Nutrient metabolism | 0.777066 | 0.059695 | 384 |
| ALL-CHORISMATE-PWY: superpathway of chorismate metabolism | Aromatic Amino Acid metabolisim superpathway | 0.92225 | 0.040834 | 382 |
| PWY-5855: ubiquinol-7 biosynthesis (prokaryotic) | Cofactor, Carrier and Vitamin metabolism | 0.728336 | 0.057305 | 382 |
| PWY-5856: ubiquinol-9 biosynthesis (prokaryotic) | Cofactor, Carrier and Vitamin metabolism | 0.728336 | 0.057305 | 382 |
| PWY-5857: ubiquinol-10 biosynthesis (prokaryotic) | Cofactor, Carrier and Vitamin metabolism | 0.728336 | 0.057305 | 382 |
| PWY-6708: ubiquinol-8 biosynthesis (prokaryotic) | Cofactor, Carrier and Vitamin metabolism | 0.728336 | 0.057305 | 382 |
| PWY-6859: all-trans-farnesol biosynthesis | Secondary Metabolite metabolism | 0.723396 | 0.061281 | 381 |
| PWY0-1338: polymyxin resistance | Lipopolysaccharide metabolism | 0.698642 | 0.069996 | 381 |
| ORNDEG-PWY: superpathway of ornithine degradation | Amine and Polyamine metabolism | 0.784056 | 0.069031 | 379 |
| PWY-5723: Rubisco shunt | Generation of Precursor Metabolites and Energy | 0.783922 | 0.067926 | 376 |
| PWY-6823: molybdenum cofactor biosynthesis | Cofactor, Carrier and Vitamin metabolism | 0.719328 | 0.076505 | 376 |
| PWY-5656: mannosylglycerate biosynthesis I | Metabolic Regulator metabolism | 0.721346 | 0.07734 | 374 |
| ARGDEG-PWY: superpathway of L-arginine, putrescine, and 4-aminobutanoate degradation | Amino Acid metabolism | 0.776694 | 0.079433 | 373 |
| ORNARGDEG-PWY: superpathway of L-arginine and L-ornithine degradation | Amino Acid metabolism | 0.776694 | 0.079433 | 373 |
| PWY-7315: dTDP-N-acetylthomosamine biosynthesis | Carbohydrate metabolism | 0.754299 | 0.079792 | 371 |
| PWY-561: superpathway of glyoxylate cycle and fatty acid degradation | Generation of Precursor Metabolites and Energy | 0.666929 | 0.071842 | 370 |
| KDO-NAGLIPASYN-PWY: superpathway of (Kdo)2-lipid A biosynthesis | Lipopolysaccharide metabolism | 0.666078 | 0.08694 | 368 |
| PWY-7392: taxadiene biosynthesis (engineered) | Secondary Metabolite metabolism | 0.755526 | 0.076004 | 367 |
| PWY-7446: sulfoglycolysis | Secondary Metabolite metabolism | 0.697943 | 0.098617 | 365 |
| PWY-6892: thiazole biosynthesis I (E. coli) | Cofactor, Carrier and Vitamin metabolism | 0.560129 | 0.09555 | 364 |
| PWY0-1415: superpathway of heme biosynthesis from uroporphyrinogen-III | Cofactor, Carrier and Vitamin metabolism | 0.607726 | 0.100936 | 360 |
| PWY-7254: TCA cycle VII (acetate-producers) | Generation of Precursor Metabolites and Energy | 0.5593 | 0.107024 | 357 |
| POLYISOPRENSYN-PWY: polyisoprenoid biosynthesis (E. coli) | Polyprenyl metabolism | 0.549328 | 0.11647 | 356 |
| P105-PWY: TCA cycle IV (2-oxoglutarate decarboxylase) | Generation of Precursor Metabolites and Energy | 0.611642 | 0.10884 | 354 |
| THISYN-PWY: superpathway of thiamin diphosphate biosynthesis I | Cofactor, Carrier and Vitamin metabolism | 0.579406 | 0.108042 | 351 |
| FERMENTATION-PWY: mixed acid fermentation | Generation of Precursor Metabolites and Energy | 0.439825 | 0.093038 | 350 |
| PWY-7409: phospholipid remodeling (phosphatidylethanolamine, yeast) | Lipids and Fatty acids metabolism | 0.640981 | 0.124214 | 348 |
| GLUCOSE1PMETAB-PWY: glucose and glucose-1-phosphate degradation | Carbohydrate metabolism | 0.466082 | 0.165896 | 340 |
| TCA-GLYOX-BYPASS: superpathway of glyoxylate bypass and TCA | Generation of Precursor Metabolites and Energy | 0.579886 | 0.130017 | 337 |
| PWY-4702: phytate degradation I | Secondary Metabolite metabolism | 0.604736 | 0.12342 | 334 |

**Table S2.** Demographic characteristics of study population based on mode of delivery and early feeding practices.

|  | **Vaginal delivery** | | **C-section with BM diet** | | **C-section with IF diet** | | ***P***^a^ |
| --- | --- | --- | --- | --- | --- | --- | --- |
|  | (n=584) | | (n=281) | | (n=167) | |  |
| Sex, N (%) |  |  |  |  |  |  | 0.765 |
| Male | 342 | (58.6) | 159 | (56.6) | 100 | (59.9) |  |
| Female | 242 | (41.4) | 122 | (43.4) | 67 | (40.1) |  |
| Age, median (IQR), mo | 64.7 | (56.0, 71.3) | 61.5 | (53.7, 70.7) | 61.8 | (53.9, 71.7) | **0.021** |
| Gestational age at birth, median (IQR), wk | 39 | (38, 40) | 39 | (38, 40) | 38 | (38, 39) | **0.001** |
| Birth Weight Z-score, median (IQR) | 0.06 | (-0.60, 0.63) | 0.28 | (-0.42, 0.91) | 0.29 | (-0.33, 0.93) | 0.059 |
| BMI for age Z-score, median (IQR), | -0.15 | (-0.89, 0.74) | 0.11 | (-0.68, 1.01) | -0.09 | (-0.78, 0.85) | **0.033** |
| BMI group, N (%) |  |  |  |  |  |  | 0.104 |
| Normal weight | 474 | (81.2) | 210 | (74.7) | 131 | (78.4) |  |
| Overweight or obese | 110 | (18.8) | 71 | (25.3) | 36 | (21.6) |  |

^a^ Statistically significant differences were determined using Kruskal-Wallis test.

**Table S3.** The impact of early feeding practices on cesareans section induced microbial compositional and functional alterations in all study participants.

|  | **Vaginal delivery** | **C-section**  **with BM diet** | **C-section**  **with IF diet** | ***P***^a^  Vaginal delivery  vs.  C-section with BM diet | ***P***^a^  Vaginal delivery  vs.  C-section with IF diet | ***P***^a^  C-section with BM diet vs.  C-section  with IF diet |
| --- | --- | --- | --- | --- | --- | --- |
|  | (n=584) | (n=281) | (n=167) |  |  |  |
| **Microbial diversity indices** |  |  |  |  |  |  |
| Shannon index | 2.64 | 2.64 | 2.65 | 1 | 1 | 1 |
| Observed Species | 103 | 102 | 96 | 0.579 | **0.001** | **0.004** |
| Simpson index | 0.872 | 0.872 | 0.875 | 0.950 | 0.930 | 0.930 |
| J Pielou index | 0.576 | 0.574 | 0.581 | 0.870 | 0.450 | 0.450 |
| **Relative abundance of microbial taxa** | | | | | | |
| ***Genus level*** |  |  |  |  |  |  |
| *Anaerostipes* | 0.010 | 0.014 | 0.017 | **0.009** | **0.003** | 0.344 |
| *Prevotella* | 0.023 | 0.016 | 0.014 | **0.021** | **0.009** | 0.421 |
| *Dorea* | 0.071 | 0.059 | 0.063 | **0.028** | **0.028** | 0.804 |
| *Clostridium* | 0.911 | 1.13 | 1.32 | **0.019** | **0.004** | 0.351 |
| *Coprobacillus* | 0.022 | 0.044 | 0.065 | 0.066 | **0.012** | 0.255 |
| *Coprococcus* $^$ | 0.215 | 0.131 | 0.130 | 0.111 | **0.007** | 0.135 |
| ***Species level*** |  |  |  |  |  |  |
| *Dorea formicigenerans* | 0.033 | 0.008 | 0.011 | **<0.0001** | **0.006** | 0.587 |
| *Dorea longicatena* | 0.009 | 0 | 0 | **0.005** | **<0.0001** | 0.051 |
| *Clostridium ramosum* | 0 | 0.003 | 0.005 | **0.002** | **0.0001** | 0.372 |
| *Clostridium bolteae* | 0.127 | 0.167 | 0.208 | **0.024** | **0.005** | 0.360 |
| *Coprobacillus unclassified* | 0.012 | 0.028 | 0.047 | **0.021** | **0.013** | 0.416 |
| *Ruminococcus gnavus* | 0.292 | 0.411 | 0.463 | **0.019** | **0.009** | 0.451 |
| *Clostridium symbiosum* | 0.041 | 0.055 | 0.069 | **0.044** | **0.012** | 0.311 |
| *Clostridium clostridioforme* | 0.002 | 0.010 | 0.003 | **0.0001** | 0.184 | 0.184 |
| *Parabacteroides distasonis* | 0.175 | 0.262 | 0.390 | **0.042** | **0.010** | 0.220 |
| *Alistipes_finegoldii* $^$ | 0.286 | 0.201 | 0.221 | **0.024** | **0.010** | 0.329 |
| **Absolute gene counts of functioning pathways** | | | | | | |
| GLUCONEO-PWY: Gluconeogenesis I | 512 | 466 | 207 | 0.264 | **0.0023** | 0.051 |
| CRNFORCAT-PWY: Creatinine degradation I | 19.3 | 0 | 0 | 0.377 | **0.0001** | **0.018** |
| PWY66-398: TCA cycle III (animals) | 30.9 | 20.7 | 5.94 | 0.056 | **<0.0001** | **0.044** |
| PWY-6590: Superpathway of Clostridium acetobutylicum acidogenic fermentation | 136 | 102 | 72.4 | 0.108 | **0.0001** | 0.075 |
| PWY-6284: Superpathway of unsaturated fatty acid biosynthesis (E.coli) | 140 | 81.4 | 66 | 0.107 | **0.023** | 0.338 |
| PWY-6113: Superpathway of mycolate biosynthesis | 240 | 157 | 107 | 0.060 | **0.014** | 0.357 |
| PWY-6285: Superpathway of fatty acid biosynthesis (E.coli) | 161 | 91.1 | 72.5 | 0.066 | **0.023** | 0.400 |
| ALL-CHORISMATE-PWY: Superpathway of chorismite metabolism $^$ | 41.8 | 31 | 15.5 | **0.036** | **0.016** | 0.371 |
| CENTEFERM-PWY: Pyruvate fermentation to butanoate | 108 | 80.3 | 56.8 | 0.107 | **0.001** | 0.075 |

Abbreviations: BM: predominant breast milk feeding; BMI: body mass index; IF: predominantly infant formula feeding.

^a^ Taxonomical profile and pathway abundancy of gut microbes are expressed as median (except where$^$were expressed as mean), statistical comparisons were performed with Wilcoxon test.

**Table S4.** The impact of early feeding practices on cesareans section induced microbial compositional and functional alterations for children under 5 years of age (<60 months).

|  | **Vaginal BM** | **Vaginal IF** | **CS**  **BM** | **CS**  **IF** | ***P***^†^  Vaginal BM  vs.  Vaginal IF | ***P***^†^  Vaginal BM  vs.  CS BM | ***P***^†^  Vaginal BM  vs.  CS IF | ***P***^†^  Vaginal IF  vs.  CS BM | ***P***^†^  Vaginal IF  vs.  CS IF | ***P***^†^  CS BM  vs.  CS IF |
| --- | --- | --- | --- | --- | --- | --- | --- | --- | --- | --- |
|  | (n=154) | (n=56) | (n=129) | (n=74) |  |  |  |  |  |  |
| **Microbial diversity indices** | | |  |  |  |  |  |  |  |  |
| Shannon index | 2.63 | 2.60 | 2.59 | 2.61 | 0.97 | 0.97 | 0.97 | 0.97 | 0.97 | 0.97 |
| Observed Species | 100 | 100 | 99 | 93 | 0.78 | 0.78 | 0.16 | 0.86 | 0.35 | 0.18 |
| Simpson index | 0.870 | 0.866 | 0.869 | 0.866 | 0.99 | 0.99 | 0.99 | 0.99 | 0.99 | 0.99 |
| J Pielou index | 0.571 | 0.571 | 0.568 | 0.591 | 0.83 | 0.83 | 0.83 | 0.83 | 0.83 | 0.83 |
| **Relative abundance of microbial taxa** | | |  |  |  |  |  |  |  |  |
| ***Genus level*** |  |  |  |  |  |  |  |  |  |  |
| *Anaerostipes* | 0.083 | 0.031 | 0.069 | 0.078 | 0.68 | **0.03** | **0.009** | 0.23 | **0.05** | 0.27 |
| *Prevotella* | 5.29 | 10.0 | 5.39 | 2.01 | 0.30 | **0.04** | **0.03** | **0.03** | **0.03** | 0.58 |
| *Dorea* | 0.186 | 0.119 | 0.128 | 0.100 | 0.22 | 0.45 | 0.12 | 0.45 | 0.67 | 0.22 |
| *Clostridium* | 2.26 | 1.81 | 2.05 | 2.24 | 0.56 | 0.77 | **0.05** | 0.58 | **0.05** | **0.05** |
| *Coprobacillus* | 0.185 | 0.197 | 0.271 | 0.352 | 0.65 | 0.75 | 0.26 | 0.65 | 0.26 | 0.26 |
| *Coprococcus*^‡^ | 0.151 | 0.102 | 0.100 | 0.144 | 0.85 | 0.85 | 0.85 | 0.85 | 0.85 | 0.85 |
| ***Species level*** |  |  |  |  |  |  |  |  |  |  |
| *Dorea formicigenerans* | 0.121 | 0.080 | 0.060 | 0.062 | 0.40 | **0.02** | **0.02** | 0.26 | 0.26 | 0.76 |
| *Dorea longicatena* | 0.051 | 0.020 | 0.042 | 0.020 | **0.04** | 0.19 | **0.0004** | 0.19 | 0.18 | **0.007** |
| *Clostridium ramosum* | 0.017 | 0.027 | 0.027 | 0.028 | 0.96 | 0.53 | 0.22 | 0.58 | 0.33 | 0.44 |
| *Clostridium bolteae* | 0.400 | 0.411 | 0.543 | 0.525 | 0.87 | 0.87 | 0.21 | 0.87 | 0.21 | 0.21 |
| *Coprobacillus unclassified* | 0.139 | 0.132 | 0.238 | 0.283 | 0.45 | 0.45 | 0.35 | 0.35 | 0.35 | 0.45 |
| *Ruminococcus gnavus* | 1.22 | 0.861 | 1.41 | 1.31 | 0.75 | 0.75 | 0.36 | 0.75 | 0.36 | 0.48 |
| *Clostridium symbiosum* | 0.148 | 0.170 | 0.134 | 0.138 | 0.97 | 0.97 | 0.44 | 0.97 | 0.56 | 0.44 |
| *Clostridium clostridioforme* | 0.089 | 0.082 | 0.088 | 0.085 | 0.97 | 0.45 | 0.45 | 0.45 | 0.45 | 0.97 |
| *Parabacteroides distasonis* | 0.826 | 0.972 | 1.04 | 1.62 | 0.56 | 0.33 | 0.33 | 0.71 | 0.56 | 0.56 |
| *Alistipes_finegoldii*^‡^ | 0.183 | 0.181 | 0.195 | 0.158 | 0.60 | 0.69 | 0.28 | 0.69 | 0.23 | 0.27 |
| **Absolute gene counts of functioning pathways** | | | |  |  |  |  |  |  |  |
| GLUCONEO-PWY: Gluconeogenesis I | 959 | 655 | 906 | 624 | 0.36 | 0.53 | 0.18 | 0.53 | 0.53 | 0.36 |
| CRNFORCAT-PWY: Creatinine degradation I | 44.8 | 30.4 | 43.6 | 27.9 | 0.17 | 0.52 | 0.17 | 0.40 | 0.99 | 0.40 |
| PWY66-398: TCA cycle III (animals) | 94.5 | 58.9 | 67.0 | 53.6 | 0.29 | 0.29 | 0.13 | 0.75 | 0.49 | 0.42 |
| PWY-6590: Superpathway of Clostridium acetobutylicum acidogenic fermentation | 190 | 136 | 189 | 116 | 0.23 | 0.40 | **0.01** | 0.569 | 0.29 | 0.18 |
| PWY-6284: Superpathway of unsaturated fatty acid biosynthesis (E.coli) | 271 | 245 | 320 | 191 | 0.34 | 0.34 | **0.03** | 0.90 | 0.34 | 0.34 |
| PWY-6113: Superpathway of mycolate biosynthesis | 431 | 397 | 448 | 262 | 0.38 | 0.27 | **0.02** | 0.93 | 0.27 | 0.27 |
| PWY-6285: Superpathway of fatty acid biosynthesis (E.coli) | 328 | 302 | 301 | 187 | 0.38 | 0.16 | **0.03** | 0.65 | 0.35 | 0.38 |
| ALL-CHORISMATE-PWY: Superpathway of chorismite metabolism | 32.5 | 35.7 | 37.6 | 18.1 | 0.24 | 0.97 | 0.97 | 0.24 | 0.24 | 0.97 |
| CENTEFERM-PWY: Pyruvate fermentation to butanoate | 151 | 108 | 151 | 92.3 | 0.24 | 0.40 | **0.01** | 0.57 | 0.30 | 0.18 |

Abbreviations: BM: predominant breast milk feeding; BMI: body mass index; IF: predominantly infant formula feeding.

^†^Taxonomical profile and pathway abundancy of gut microbes are expressed as mean; statistical comparisons were performed with Wilcoxon test.

**Table S5.**The impact of early feeding practices on cesareans section induced microbial compositional and functional alterations for children aged five ($\geq$60, <72 months).

|  | **Vaginal BM** | **Vaginal IF** | **CS**  **BM** | **CS**  **IF** | ***P***^†^  Vaginal BM  vs.  Vaginal IF | ***P***^†^  Vaginal BM  vs.  CS BM | ***P***^†^  Vaginal BM  vs.  CS IF | ***P***^†^  Vaginal IF  vs.  CS BM | ***P***^†^  Vaginal IF  vs.  CS IF | ***P***^†^  CS BM  vs.  CS IF |
| --- | --- | --- | --- | --- | --- | --- | --- | --- | --- | --- |
|  | (n=167) | (n=70) | (n=92) | (n=54) |  |  |  |  |  |  |
| **Microbial diversity indices** | | | |  |  |  |  |  |  |  |
| Shannon index | 2.62 | 2.68 | 2.61 | 2.69 | 0.68 | 0.68 | 0.68 | 0.68 | 0.90 | 0.68 |
| Observed Species | 104 | 100 | 103 | 97 | 0.25 | 0.67 | 0.19 | 0.43 | 0.66 | 0.25 |
| Simpson index | 0.872 | 0.882 | 0.867 | 0.885 | 0.58 | 0.87 | 0.54 | 0.58 | 0.69 | 0.54 |
| J Pielou index | 0.577 | 0.588 | 0.566 | 0.582 | 0.39 | 0.78 | 0.34 | 0.34 | 0.79 | 0.34 |
| **Relative abundance of microbial taxa** | | | |  |  |  |  |  |  |  |
| ***Genus level*** |  |  |  |  |  |  |  |  |  |  |
| *Anaerostipes* | 0.043 | 0.063 | 0.057 | 0.128 | 0.96 | 0.60 | 0.60 | 0.60 | 0.60 | 0.96 |
| *Prevotella* | 7.78 | 7.05 | 6.48 | 4.60 | 0.79 | 0.90 | 0.54 | 0.79 | 0.54 | 0.54 |
| *Dorea* | 0.156 | 0.238 | 0.118 | 0.095 | 0.77 | **0.003** | 0.1231 | **0.01** | 0.13 | 0.26 |
| *Clostridium* | 1.81 | 1.89 | 2.98 | 2.43 | 0.40 | **0.04** | 0.187 | 0.28 | 0.47 | 0.74 |
| *Coprobacillus* | 0.126 | 0.171 | 0.311 | 0.328 | 0.42 | 0.11 | 0.11 | 0.42 | 0.42 | 0.85 |
| *Coprococcus*^‡^ | 0.261 | 0.250 | 0.143 | 0.117 | 0.29 | 0.29 | 0.07 | 0.84 | 0.29 | 0.29 |
| ***Species level*** |  |  |  |  |  |  |  |  |  |  |
| *Dorea formicigenerans* | 0.085 | 0.087 | 0.052 | 0.053 | 0.85 | **0.05** | 0.16 | 0.15 | 0.25 | 0.82 |
| *Dorea longicatena* | 0.058 | 0.131 | 0.054 | 0.026 | 0.97 | **0.02** | **0.04** | **0.04** | 0.10 | 0.97 |
| *Clostridium ramosum* | 0.013 | 0.012 | 0.023 | 0.033 | 0.93 | 0.06 | 0.14 | 0.136 | 0.20 | 0.99 |
| *Clostridium bolteae* | 0.357 | 0.475 | 0.865 | 0.593 | 0.46 | 0.16 | 0.16 | 0.46 | 0.46 | 0.90 |
| *Coprobacillus unclassified* | 0.111 | 0.099 | 0.184 | 0.264 | 0.76 | 0.16 | 0.16 | 0.16 | 0.16 | 0.76 |
| *Ruminococcus gnavus* | 0.830 | 0.843 | 1.12 | 1.42 | 0.84 | **0.05** | 0.12 | **0.05** | 0.11 | 0.83 |
| *Clostridium symbiosum* | 0.137 | 0.145 | 0.169 | 0.203 | 0.46 | 0.06 | 0.34 | 0.46 | 0.67 | 0.67 |
| *Clostridium clostridioforme* | 0.116 | 0.027 | 0.088 | 0.060 | 0.73 | **0.03** | 0.49 | 0.13 | 0.73 | 0.37 |
| *Parabacteroides distasonis* | 0.742 | 0.757 | 0.965 | 1.10 | 0.43 | 0.30 | 0.08 | 0.60 | 0.30 | 0.39 |
| *Alistipes_finegoldii*^‡^ | 0.431 | 0.318 | 0.259 | 0.391 | 0.39 | **0.03** | 0.39 | 0.39 | 1.00 | 0.39 |
| **Absolute gene counts of functioning pathways** | | | |  |  |  |  |  |  |  |
| GLUCONEO-PWY: Gluconeogenesis I | 956 | 756 | 825 | 711 | 0.14 | 0.23 | 0.14 | 0.83 | 0.83 | 0.63 |
| CRNFORCAT-PWY: Creatinine degradation I | 51.6 | 37.9 | 51.4 | 30.3 | 0.12 | 0.51 | **0.02** | 0.356 | 0.36 | 0.11 |
| PWY66-398: TCA cycle III (animals) | 90.4 | 108 | 95.5 | 37.1 | 0.53 | 0.50 | **0.02** | 0.426 | **0.02** | 0.13 |
| PWY-6590: Superpathway of Clostridium acetobutylicum acidogenic fermentation | 211 | 157 | 149 | 178 | 0.29 | 0.14 | 0.35 | 0.92 | 0.92 | 0.92 |
| PWY-6284: Superpathway of unsaturated fatty acid biosynthesis (E.coli) | 272 | 339 | 252 | 186 | 0.46 | 0.46 | 0.32 | 0.32 | 0.26 | 0.46 |
| PWY-6113: Superpathway of mycolate biosynthesis | 415 | 520 | 385 | 284 | 0.49 | 0.49 | 0.31 | 0.31 | 0.31 | 0.49 |
| PWY-6285: Superpathway of fatty acid biosynthesis (E.coli) | 301 | 363 | 290 | 190 | 0.34 | 0.70 | 0.31 | 0.34 | 0.15 | 0.34 |
| ALL-CHORISMATE-PWY: Superpathway of chorismite metabolism | 37.7 | 51.9 | 30.2 | 15.1 | 0.82 | 0.71 | 0.35 | 0.82 | 0.44 | 0.44 |
| CENTEFERM-PWY: Pyruvate fermentation to butanoate | 169 | 125 | 118 | 142 | 0.31 | 0.13 | 0.36 | 0.93 | 0.93 | 0.93 |

Abbreviations: BM: predominant breast milk feeding; BMI: body mass index; IF: predominantly infant formula feeding.

^†^Taxonomical profile and pathway abundancy of gut microbes are expressed as mean; statistical comparisons were performed with Wilcoxon test.

**Table S6.** The impact of early feeding practices on cesareans section induced microbial compositional and functional alterations for children age above five years old ($\geq$72 months).

|  | **Vaginal BM** | **Vaginal IF** | **CS**  **BM** | **CS**  **IF** | ***P***^†^  Vaginal BM  vs.  Vaginal IF | ***P***^†^  Vaginal BM  vs.  CS BM | ***P***^†^  Vaginal BM  vs.  CS IF | ***P***^†^  Vaginal IF  vs.  CS BM | ***P***^†^  Vaginal IF  vs.  CS IF | ***P***^†^  CS BM  vs.  CS IF |
| --- | --- | --- | --- | --- | --- | --- | --- | --- | --- | --- |
|  | (n=98) | (n=35) | (n=60) | (n=39) |  |  |  |  |  |  |
| **Microbial diversity indices** | | | |  |  |  |  |  |  |  |
| Shannon index | 2.67 | 2.67 | 2.73 | 2.61 | 0.59 | 0.59 | 0.81 | 0.90 | 0.59 | 0.59 |
| Observed Species | 105 | 102 | 104 | 94 | 0.71 | 0.71 | 0.14 | 0.99 | 0.29 | 0.29 |
| Simpson index | 0.870 | 0.880 | 0.889 | 0.875 | 0.59 | 0.59 | 0.79 | 0.79 | 0.59 | 0.59 |
| J Pielou index | 0.577 | 0.591 | 0.594 | 0.577 | 0.57 | 0.57 | 0.93 | 0.93 | 0.65 | 0.57 |
| **Relative abundance of microbial taxa** | | | |  |  |  |  |  |  |  |
| ***Genus level*** |  |  |  |  |  |  |  |  |  |  |
| *Anaerostipes* | 0.029 | 0.031 | 0.114 | 0.049 | 0.8 | 0.8 | 0.8 | 0.8 | 0.8 | 0.8 |
| *Prevotella* | 5.63 | 6.31 | 4.36 | 6.13 | 0.8 | 0.8 | 0.8 | 0.8 | 0.8 | 0.8 |
| *Dorea* | 0.161 | 0.178 | 0.147 | 0.155 | 0.8 | 0.8 | 0.8 | 0.8 | 0.8 | 0.8 |
| *Clostridium* | 1.76 | 1.56 | 1.94 | 1.68 | 0.84 | 0.54 | 0.59 | 0.54 | 0.59 | 0.59 |
| *Coprobacillus* | 0.253 | 0.276 | 0.180 | 0.123 | 0.59 | 0.71 | 0.97 | 0.71 | 0.71 | 0.71 |
| *Coprococcus*^‡^ | 0.160 | 0.114 | 0.165 | 0.099 | 0.78 | 0.78 | 0.77 | 0.90 | 0.78 | 0.78 |
| ***Species level*** |  |  |  |  |  |  |  |  |  |  |
| *Dorea formicigenerans* | 0.096 | 0.075 | 0.074 | 0.086 | 0.97 | 0.52 | 0.97 | 0.52 | 0.97 | 0.71 |
| *Dorea longicatena* | 0.053 | 0.094 | 0.043 | 0.057 | 0.36 | 0.59 | 0.63 | 0.36 | 0.36 | 0.93 |
| *Clostridium ramosum* | 0.018 | 0.012 | 0.018 | 0.015 | 0.38 | 0.50 | 0.38 | 0.30 | 0.30 | 0.76 |
| *Clostridium bolteae* | 0.533 | 0.395 | 0.482 | 0.451 | 0.896 | 0.048 | 0.439 | 0.138 | 0.528 | 0.566 |
| *Coprobacillus unclassified* | 0.122 | 0.054 | 0.074 | 0.071 | 0.78 | 0.97 | 0.78 | 0.78 | 0.78 | 0.78 |
| *Ruminococcus gnavus* | 0.883 | 0.810 | 0.924 | 0.775 | 0.98 | 0.98 | 0.98 | 0.98 | 0.98 | 0.98 |
| *Clostridium symbiosum* | 0.098 | 0.113 | 0.143 | 0.194 | 0.36 | 0.36 | 0.20 | 0.71 | 0.71 | 0.57 |
| *Clostridium clostridioforme* | 0.052 | 0.065 | 0.089 | 0.060 | 0.54 | 0.18 | 0.78 | 0.70 | 0.78 | 0.54 |
| *Parabacteroides distasonis* | 1.23 | 0.604 | 1.16 | 1.18 | 0.42 | 0.75 | 0.68 | 0.37 | 0.37 | 0.75 |
| *Alistipes_finegoldii*^‡^ | 0.263 | 0.217 | 0.126 | 0.107 | 0.80 | 0.27 | 0.14 | 0.45 | 0.27 | 0.45 |
| **Absolute gene counts of functioning pathways** | | | |  |  |  |  |  |  |  |
| GLUCONEO-PWY: Gluconeogenesis I | 1089 | 831 | 968 | 617 | 0.56 | 0.57 | 0.10 | 0.84 | 0.43 | 0.40 |
| CRNFORCAT-PWY: Creatinine degradation I | 65.6 | 46.1 | 56.6 | 32.2 | 0.38 | 0.38 | 0.12 | 0.83 | 0.38 | 0.38 |
| PWY66-398: TCA cycle III (animals) | 75.6 | 84.5 | 64.2 | 46.3 | 0.66 | 0.49 | 0.28 | 0.49 | 0.28 | 0.50 |
| PWY-6590: Superpathway of Clostridium acetobutylicum acidogenic fermentation | 258 | 178 | 226 | 142 | 0.46 | 0.65 | 0.13 | 0.46 | 0.46 | 0.20 |
| PWY-6284: Superpathway of unsaturated fatty acid biosynthesis (E.coli) | 342 | 358 | 246 | 273 | 0.82 | 0.82 | 0.85 | 0.82 | 0.84 | 0.82 |
| PWY-6113: Superpathway of mycolate biosynthesis | 479 | 484 | 358 | 409 | 0.68 | 0.68 | 0.76 | 0.68 | 0.76 | 0.68 |
| PWY-6285: Superpathway of fatty acid biosynthesis (E.coli) | 332 | 366 | 245 | 333 | 0.70 | 0.70 | 0.70 | 0.70 | 0.77 | 0.70 |
| ALL-CHORISMATE-PWY: Superpathway of chorismite metabolism^‡^ | 54.2 | 56.3 | 17.9 | 11.2 | 0.636 | 0.052 | 0.052 | 0.442 | 0.353 | 0.642 |
| CENTEFERM-PWY: Pyruvate fermentation to butanoate | 207 | 143 | 180 | 114 | 0.45 | 0.65 | 0.13 | 0.45 | 0.45 | 0.21 |

Abbreviations: BM: predominant breast milk feeding; BMI: body mass index; IF: predominantly infant formula feeding.

^†^Taxonomical profile and pathway abundancy of gut microbes are expressed as mean; statistical comparisons were performed with Wilcoxon test.

Figure legend

**Figure S1.** Comparison of the gut microbial alpha indices (Shannon Wiener Index, Simpson index, Richness and J Pielou Index) between children born vaginally or via cesarean section in different age groups, (a)age under five (<60 months), (b) age at five ($\geq$60, $<$72 months), (c) age above five ($\geq$72 months).

**Figure S2**. Comparison of the distribution of previously identified differential microbial taxa between children born virginally and via cesarean section in stratified analysis defined by age, (a) genus level at age under five (<60 months), (b) species level at age under five (<60 months) (c) genus level age at five ($\geq$60, $<$72 months), (d) species level age at five ($\geq$60, $<$72 months), (e) genus level age above five ($\geq$72 months), (f) species level at age above five ($\geq$72 months).

**References:**

1. Villar J, Cheikh Ismail L, Victora CG, Ohuma EO, Bertino E, Altman DG, et al. International standards for newborn weight, length, and head circumference by gestational age and sex: the Newborn Cross-Sectional Study of the INTERGROWTH-21st Project. Lancet. 2014;384(9946):857-68.

2. Kuang Y-S, Lu J-H, Li S-H, Li J-H, Yuan M-Y, He J-R, et al. Connections between the human gut microbiome and gestational diabetes mellitus. Gigascience. 2017;6(8).

3. Fang C, Zhong H, Lin Y, Chen B, Han M, Ren H, et al. Assessment of the cPAS-based BGISEQ-500 platform for metagenomic sequencing. GigaScience. 2018;7(3):1-8.

4. Mandal S, Van Treuren W, White RA, Eggesbø M, Knight R, Peddada SD. Analysis of composition of microbiomes: a novel method for studying microbial composition. Microb Ecol Health Dis. 2015;26:27663.


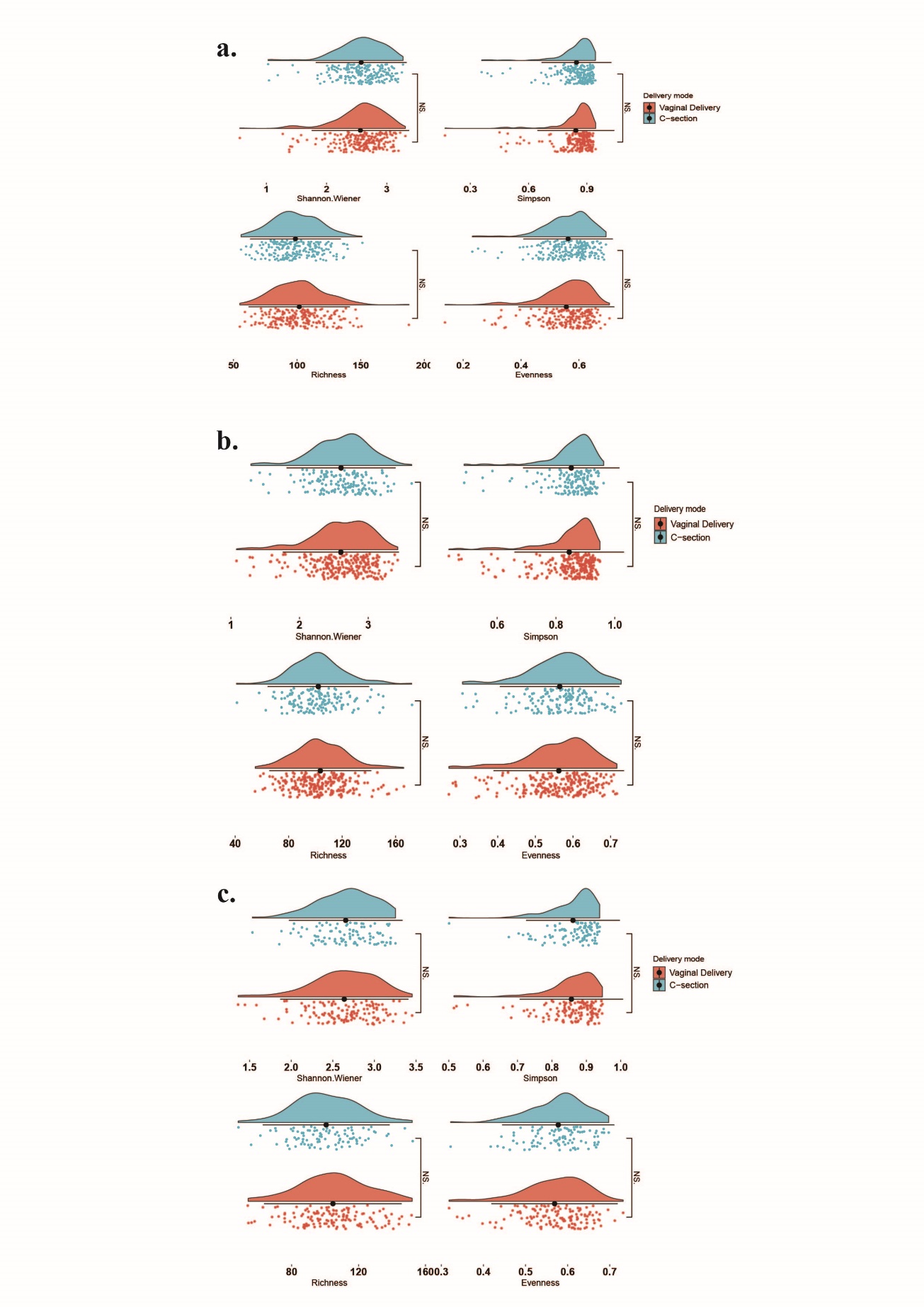


Figure S1


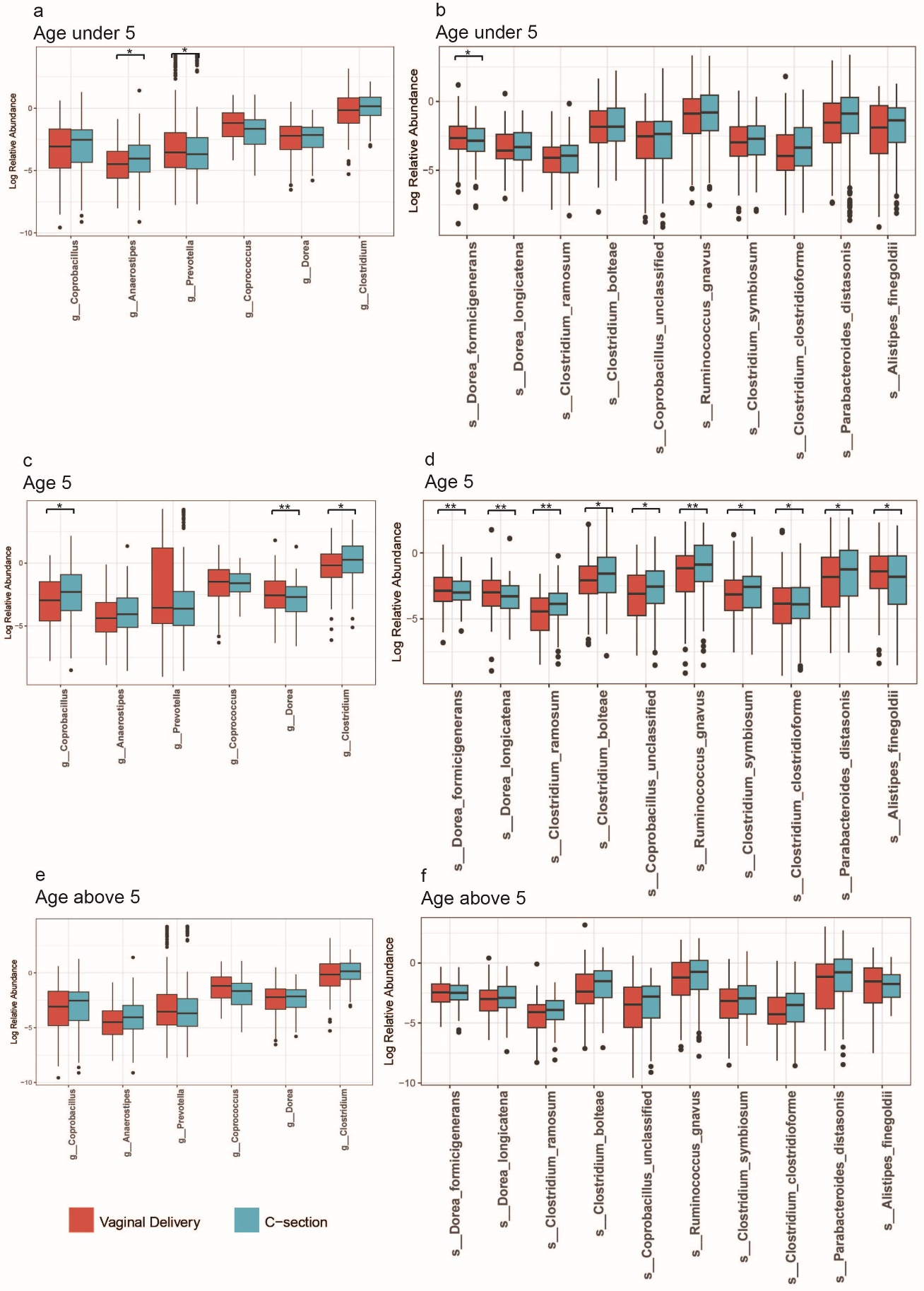


Figure S2
